# Pigmented villonodular synovitis of the knee in a patient on oral anticoagulation therapy: a case report

**DOI:** 10.1186/1752-1947-3-121

**Published:** 2009-11-13

**Authors:** Balasundaram Ramesh, Sanathkumar Shetty, Salah S Bastawrous

**Affiliations:** 1 Department of Trauma and Orthopaedics, Glan Clwyd Hospital, Sarn Lane, Bodelwyddan, Rhyl, North Wales, LL18 5UJ, UK

## Abstract

**Introduction:**

Pigmented villonodular synovitis is a disease which affects the synovial joints and tendon sheaths. Although the exact aetiological factors are not known, we believe that recurrent haemarthrosis has a role in the aetiology of this condition.

**Case presentation:**

A 62-year-old Caucasian man presented with gradually worsening pain and stiffness in his right knee. The patient was on anticoagulation therapy and had been treated for recurrent episodes of spontaneous haemarthrosis of the knee. The International Normalized Ratio on each occasion suggested poor control of the anticoagulation therapy. A diagnosis of pigmented villonodular synovitis was made based on intra-operative findings and was further confirmed by a histopathological examination.

**Conclusion:**

This report is presented to highlight the unusual association of haemarthrosis and pigmented villonodular synovitis.

## Introduction

Pigmented villonodular synovitis (PVNS) is a disease of unknown aetiology affecting the synovial joints. The aetiology of PVNS remains controversial and a number of theories have been postulated. Haemarthrosis has been suggested as a possible aetiological factor. Only one description of PVNS of the ankle in a patient on anticoagulation therapy has been previously reported [1]; we describe the second known case of PVNS of the knee joint in a patient on anticoagulation therapy.

## Case presentation

A 62-year-old Caucasian man had an uneventful total left knee replacement five years prior to presentation. Two years following the total knee replacement, the patient was diagnosed with dilated cardiomyopathy and was started on warfarin. Following this, he had recurrent episodes of sudden pain and swelling on his right knee. During each of these episodes, there was no history of trauma and the patient was systemically well. Although his International Normalized Ratio (INR) was high, his blood tests for full blood count and C-reactive protein were within the normal range. All of these episodes were in the initial phase of his warfarin therapy and they ceased once his INR was stabilized.

**Figure 1:**
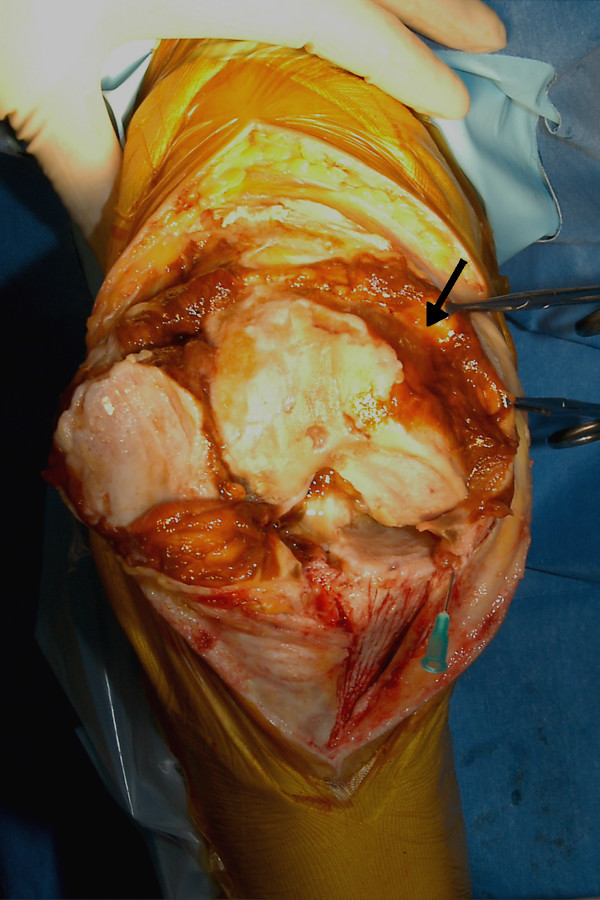
**Intra-operative photographs showing the reddish-orange stained synovium (black arrow)**.

**Figure 2:**
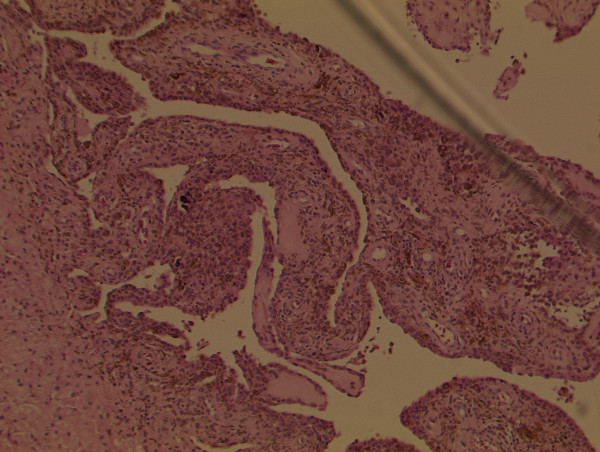
**Photomicrograph showing the presence of haemosiderin deposits, foamy histiocytes and giant cells confirming pigmented villonodular synovitis (haematoxylin and eosin stain)**.

The condition of the patient's right knee gradually deteriorated. Clinically, the knee joint was diffusely swollen and tender but stable. His active range of movement was from neutral to 100 degrees of flexion and any further flexion was painful. Radiographs of the knee showed advanced arthritic changes and he was admitted for a total knee replacement. Intraoperatively, the synovium was found to be hypertrophic and stained reddish orange and the synovial fluid was reddish-orange in colour (Figure 1). These appearances suggested a diagnosis of PVNS. A synovectomy was performed, which was then followed by a total knee replacement. A synovial specimen was sent for histopathological examination. The microscopic features were consistent with a diagnosis of PVNS (Figure 2). The postoperative period was uneventful and the patient was asymptomatic after three years of follow-up treatment.

## Discussion

PVNS typically occurs in adults in their third or fourth decade of life, with a male-to-female ratio of 1.9 to 1.3. Involvement is usually monoarticular [2]. The knee joint is the most frequently affected site, followed by the fingers, feet, ankles, hips, wrists and shoulders in a decreasing order of frequency [2,3].

Since the first description of this condition by Jaffe *et al. *in 1941 [4], the aetiology of this benign tumour involving the synovial membrane has remained unclear. Jaffe proposed that a hypervascular cellular phase occurs after trauma produc ing hyalinization and fibrosis [4]. Various aetiologies including trauma [2], inflammation [3], haemorrhage [5], neoplasia [6] and genetic factors [7] have been suggested. Chronic recurrent microtrauma and haemarthrosis have also been postulated [2].

It has also been postulated that PVNS in children arises through a different mechanism to that in adults, and it is also possible that not all lesions interpreted as PVNS share the same mechanism [8].

There are very few cases of PVNS reported in patients on anticoagulation therapy [1] and with a bleeding disorder [9]. In our patient, the symptoms in the knee worsened following these repeated episodes of haemarthrosis and the INR on these occasions showed a poor control of his anticoagulation therapy.

## Conclusion

This case supports the argument of earlier reports [1,9] that repeated haemarthrosis may have a role in the aetiology of PVNS. We hope that this study will encourage the reporting of similar cases to lead to a better understanding of the aetiology of this condition.

## Abbreviations

INR: International Normalized Ratio; PVNS: pigmented villonodular synovitis.

## Competing interests

The authors declare that they have no competing interests.

## Authors' contributions

SS made substantial contributions in acquiring data, reviewing the literature and preparing the manuscript. BR performed the knee replacement operation and also contributed in reviewing the literature and drafting the manuscript. SSB gave final approval to the draft to be published. All authors read and approved the final manuscript.

## Consent

Written informed consent was obtained from the patient for publication of this case report and any accompanying images. A copy of the written consent is available for review by the Editor-in-Chief of this journal.
